# Editorial Note: Machine learning-driven Diabetes Health Tracer (DHT): Optimizing prognosis using RaSK_GraDe and RaSK_GraDeL models

**DOI:** 10.1371/journal.pone.0346001

**Published:** 2026-03-30

**Authors:** The PLOS One Editors

The *PLOS One* Editors issue this Editorial Note to inform readers that the work cited in this article [1] as Reference 32 was retracted before the publication date of [1]. PLOS considers that this reference is not crucial in supporting the research or conclusions reported in [1], but the following sentences are no longer supported:

- Section 2 Literature Review, paragraph 15, sentence 1: “In [32], the author developed the intelligent diabetes mellitus prediction framework (IDMPF), a framework for diabetes prediction. The Pima dataset was utilised by them. The achieved accuracy was 83%. However, the model gives result but not so good, so their is a way to make it better.”

We regret that the issues were not identified prior to the article’s publication.
